# Extracellular vesicles in laboratory medicine: a review and outlook

**DOI:** 10.3389/fcell.2025.1709461

**Published:** 2025-11-06

**Authors:** Xingdong Wang, Meijin Liu, Minghong Zhao, Haibin Shen, Qing Jin, Dingyu Rao, Defa Huang

**Affiliations:** 1 Department of Pharmacy, Aerospace Center Hospital, Beijng, China; 2 Laboratory Medicine, People’s Hospital of Ganzhou Economic Development Zone, Ganzhou, China; 3 Laboratory Medicine, Guizhou Aerospace Hospital, Zunyi, China; 4 School of Pharmacy, Henan University, Kaifeng, China; 5 Laboratory Medicine, First Affiliated Hospital of Gannan Medical University, Ganzhou, China; 6 Department of Cardiothoracic Surgery, The First Affiliated Hospital of Gannan Medical University, Ganzhou, China

**Keywords:** extracellular vesicles, laboratory medicine, disease diagnosis, prognostic evaluation, development trends

## Abstract

Extracellular vesicles (EVs), serving as promising novel biomarkers for diseases, demonstrate extensive potential applications in disease diagnosis, prognosis evaluation, and treatment monitoring. Currently, EVs have made substantial advancements in the areas of disease diagnosis, prognosis, and treatment. Nevertheless, for EVs to be fully integrated into clinical laboratories, ongoing efforts are required in multi-omics integration and big data analysis, the development of clinically applicable separation and detection technologies, the establishment of standardized quality systems, as well as clinical trials and regulatory approval processes. This paper reviews the current status of the application of extracellular vesicles in disease diagnosis, prognostic assessment, and treatment monitoring, analyzes the challenges facing current research, and discusses future trends.

## 1 Introduction

Extracellular vesicles (EVs) are small membrane-bound vesicles secreted by various cell types, containing a diverse array of biomolecules, including proteins, lipids, and nucleic acids (van Niel et al., 2018; Kalluri and LeBleu, 2020). These vesicles play a crucial role in intercellular communication, facilitating the exchange of information and materials between cells (Boudreau et al., 2014; Liu and Wang, 2023). The past two decades have seen a significant surge in research focusing on the biological functions of EVs, particularly in the field of diagnostic medicine. As the understanding of EVs’ roles in various physiological and pathological processes deepens, their potential applications in clinical diagnostics and therapeutics are becoming increasingly evident.

The exploration of EVs has revealed their involvement in numerous biological processes, including immune response, tumor progression, and tissue repair (Kalluri, 2024; Marar et al., 2021; Ding et al., 2021; Zhang and Yu, 2019). Their ability to carry specific molecular signatures reflective of their parent cells makes them promising biomarkers for various diseases, including cancer, neurodegenerative disorders, and cardiovascular diseases (Chen et al., 2024a). For instance, small extracellular vesicles (sEVs) have demonstrated functional roles akin to their originating cells, minus the risk of tumorigenicity, positioning them as viable candidates for regenerative medicine and diagnostic applications (Jia et al., 2022). The integration of sEVs into clinical practice could revolutionize how diseases are diagnosed and monitored, providing non-invasive and highly specific means to assess disease states.

Despite the promising potential of EVs in diagnostic medicine, several challenges hinder their widespread application. One major obstacle is the standardization of isolation and characterization methods, which can significantly impact the reproducibility and reliability of research findings (Kumari et al., 2024; Akbar et al., 2022). Variability in techniques used to isolate EVs can lead to discrepancies in the yield and purity of vesicles, complicating their use as biomarkers (Zhang Q. et al., 2023). Furthermore, the molecular heterogeneity of EVs poses challenges in identifying specific markers that can be reliably associated with particular diseases (Mathieu et al., 2021). Addressing these issues will require collaborative efforts among researchers to develop standardized protocols and robust analytical methods to ensure the consistency and validity of EV-based diagnostics.

Looking ahead, the future of EV research in diagnostic medicine appears promising, with several trends emerging. Advances in engineering strategies, such as the development of novel biomaterials for EV delivery and targeted therapies, are likely to enhance the therapeutic applications of EVs (Urabe et al., 2020; Liang et al., 2021). Additionally, the exploration of mesenchymal stem cell-derived sEVs and their role in cartilage repair highlights a growing interest in the regenerative potential of EVs (Ju et al., 2023). As researchers continue to investigate the mechanisms underlying EV biogenesis and function, it is anticipated that new insights will pave the way for innovative diagnostic tools and therapeutic strategies, ultimately improving patient outcomes in various medical fields. This review provides an overview of the types and features of EVs and highlights their applications in diagnosing, predicting outcomes, and tracking treatment responses in cancer, autoimmune disorders, and infectious diseases. Additionally, we examined the obstacles facing EVs in laboratory medicine, highlighted their prospective development directions, and provided new perspectives to promote research related to EVs.

## 2 Classification and characteristics of extracellular vesicles

**FIGURE 1:**
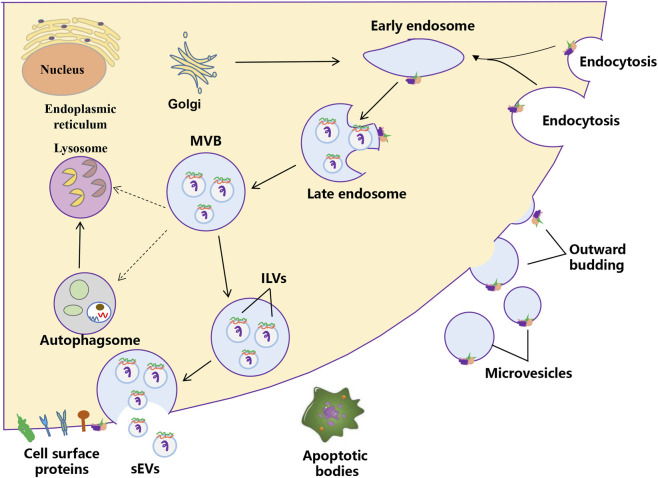
The biogenesis process of extracellular vesicles. Two mechanisms of EVs biogenesis are illustrated. The process of releasing sEVs into the extracellular milieu contains three distinct steps: sEVs biogenesis, intracellular trafficking of MVBs, and fusion of MVBs with the plasma membrane. Early endosomes are formed by the inward budding of the plasma membrane, or in some cases from the Golgi. Early endosomes mature into late endosomes and finally generate MVBs, in which process intraluminal vesicles (ILVs) are formed by inward invagination of the endosome limiting membrane. The fate of MVBs can be fusion with the plasma membrane, which results in the release of sEVs. Alternatively, MVBs can fuse with lysosomes/autophagosomes for degradation. Microvesicles arise from the direct outward budding and fission of the plasma membrane.

EVs are heterogeneous membrane-bound particles released by cells, playing crucial roles in intercellular communication and various physiological processes (Kalluri and LeBleu, 2020; Cheng et al., 2014). According to the MISEV2018 and MISEV2023 guidelines, EVs are broadly categorized into two main subtypes: small extracellular vesicles (sEVs), defined as having a diameter of less than 200 nm, and large extracellular vesicles (including microvesicles and apoptotic bodies), characterized by a diameter exceeding 200 nm (Thery et al., 2018; Welsh et al., 2024) (Figure 1). sEVs are formed through inward budding of the endosomal membrane, resulting in the formation of multivesicular bodies. These multivesicular bodies subsequently fuse with the plasma membrane, releasing intraluminal vesicles into the extracellular space (Shao et al., 2018). Microvesicles, on the other hand, are larger (200–1,000 nm) and are generated by direct outward budding from the plasma membrane (Gurunathan et al., 2019). Apoptotic bodies are even larger (1–5 µm) and are released during the process of programmed cell death, containing cellular debris and organelles (Akers et al., 2013). The classification of EVs is essential for understanding their distinct biogenesis, composition, and functional roles in health and disease (Chen et al., 2024b; Ren et al., 2024; Martinez-Garcia et al., 2024). Besides EVs, EV-like particles are increasingly gaining interest. These EV-like particles generally consist of non-vesicular protein aggregates or complexes, lipoprotein particles, viral or bacterial particles, and artificial substances generated during sample preparation (Wang et al., 2022). The process by which EV-like particles form is not completely understood and might be linked to the clustering of cellular metabolites or particular cellular activities. Certain EV-like particles carry specific biomarkers and could play a role in disease diagnosis; however, their functions are quite complex and need more research.

sEVs are defined as nanoscale vesicles that originate from the endosomal system of cells. Their biogenesis begins with the inward budding of the endosomal membrane, forming intraluminal vesicles (ILVs) within multivesicular bodies (MVBs). When these MVBs fuse with the plasma membrane, sEVs are released into the extracellular environment. This process is regulated by various proteins, including members of the ESCRT (endosomal sorting complexes required for transport) machinery, which facilitate the sorting of specific cargo into sEVs (van Niel et al., 2018; Kowal et al., 2014; Ostrowski et al., 2010). The cargo of sEVs is diverse, which can influence the behavior of recipient cells. The unique composition of sEVs reflects the physiological state of their parent cells, making them valuable biomarkers for various diseases, including cancer (Park et al., 2023; Lai et al., 2023).

Microvesicles and apoptotic bodies represent two distinct types of extracellular vesicles, each with unique characteristics and functions. Microvesicles are formed through the outward budding of the plasma membrane and are typically larger than sEVs, ranging from 200 to 1,000 nm (Wang et al., 2023). They contain a variety of bioactive molecules and are involved in processes such as cell signaling, inflammation, and tissue repair. In contrast, apoptotic bodies are released during the process of apoptosis, the programmed cell death that occurs in response to cellular stress or damage. They are larger (1–5 µm) and contain cellular debris, including organelles and fragments of the cytoplasm. Unlike microvesicles, which can be involved in intercellular communication, apoptotic bodies primarily serve as a mechanism for the removal of dying cells and their components from the body. The distinct biogenesis, size, and contents of these vesicles underscore their different roles in cellular processes and disease mechanisms (Zhang et al., 2024).

EVs play a multifaceted and critical role in intercellular communication, significantly influencing a wide range of physiological and pathological processes. They enable the transfer of proteins, lipids, and genetic material between cells, thereby modulating signaling pathways that affect cellular behavior, immune responses, and tissue regeneration. For example, sEVs derived from stem cells have demonstrated potential in facilitating tissue repair and regeneration across various injury models (Trubiani et al., 2019). Moreover, EVs are involved in disease progression, particularly in cancer, where tumor-derived extracellular vesicles can alter the tumor microenvironment, promote metastasis, and facilitate immune evasion (Liu et al., 2025). The capacity of EVs to transport specific molecular cargoes, such as microRNAs and proteins, renders them promising candidates as biomarkers for disease diagnosis and prognosis (Kumar et al., 2024; De Giorgis et al., 2024; He et al., 2024). Their pivotal role in mediating cell-to-cell communication underscores their potential as therapeutic agents in regenerative medicine and targeted drug delivery systems (Ramesh et al., 2023).

## 3 Applications of extracellular vesicles in disease diagnosis

**FIGURE 2:**
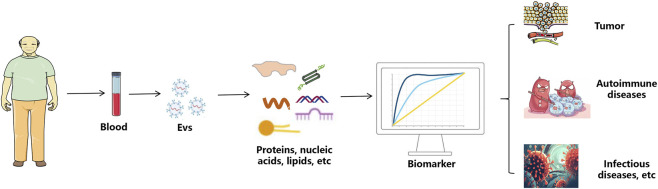
The application process of extracellular vesicles in disease diagnosis. sEVs are isolated from the body’s blood. The contents of proteins, nucleic acids and lipids in sEVs can be used for the diagnosis of tumor, autoimmune diseases and infectious diseases.

Liquid biopsy, an advanced technology for analyzing body fluid samples, is gaining traction in cancer diagnostics and monitoring. Blood-based liquid biopsy, particularly focusing on cell-free DNAs (cf-DNAs), circulating tumor cells (CTCs), and EVs, has garnered significant attention (Su et al., 2024). The benefits of analyzing EVs through liquid biopsy include: (1) EVs are found in higher concentrations in bodily fluids compared to circulating tumor cells; (2) EVs offer more detailed information about the cells that produce them than circulating DNA does; and (3) EVs exhibit strong biological stability even within the harsh tumor environment (Kalluri and LeBleu, 2020; Yu et al., 2022; Mathew et al., 2020). The potential of EVs in clinical diagnostics is vast, spanning across tumor detection, autoimmune diseases, and infectious diseases (Figure 2). Their non-invasive nature allows for the collection of biological fluids such as blood, urine, and saliva, making them ideal candidates for liquid biopsies. This section will explore the specific applications of EVs in identifying tumor markers, diagnosing autoimmune diseases early, and detecting biomarkers for infectious diseases.

The identification of tumor markers *via* the analysis of EVs has transformed cancer diagnostics (Youssef et al., 2025). Tumor-derived EVs (tEVs) contain a diverse array of biomolecules, such as proteins, lipids, and nucleic acids, which offer valuable insights into tumor presence and progression. For example, research has demonstrated that elevated levels of specific proteins, including Glypican-3 (GPC3), in circulating EVs are correlated with hepatocellular carcinoma, establishing them as reliable indicators for early detection (Qu et al., 2023). Based on microfluidic technology with fine microstructures and precise microfluidic operations, this dPCR chip enables accurate quantification of tumor-derived extracellular vesicles (SEVs) across various tumor markers, demonstrating exceptional sensitivity (detection limit: 10 copies). In clinical sample analysis, the chip effectively distinguishes lung cancer patients from healthy controls (P < 0.001, two-tailed t-test). Furthermore, in samples with extremely low target concentrations, it exhibits significantly superior quantitative accuracy compared to quantitative real-time polymerase chain reaction (qPCR). Furthermore, circulating EVs have shown potential in monitoring metastasis in breast cancer patients, with significant differences in tEV levels observed between metastatic and non-metastatic cases (Xu et al., 2024). Pancreatic cancer (PC) is a highly aggressive digestive system cancer, with pancreatic ductal adenocarcinoma (PDAC) representing about 90% of all PC cases. Xu and colleagues created a diagnostic model using a set of biomarkers, including three types of miRNAs and CA19-9, which achieved an AUC of 0.97, sensitivity of 0.95, and specificity of 0.96 (Nakamura et al., 2022). Melo and his team discovered that identifying glypican-1 in EVs among pancreatic cancer (PC) patients showed perfect sensitivity and specificity (100%) in diagnosing all stages of PC, effectively differentiating pancreatic cancer patients from healthy individuals or those with chronic pancreatitis (AUC = 1.0) (Melo et al., 2015). This characteristic positions EVs as promising candidates for liquid biopsy applications, offering a non-invasive alternative to traditional tissue biopsies and facilitating real-time monitoring of tumor dynamics. At present, the application of EVs in clinical trials for tumor diagnosis is gradually increasing. LIVER-TRACK aims at reliably predicting the outcome of patients with compensated cirrhosis through the development of a Tests for Decompensation and a Test for HCC. This will be achieved through leveraging circulating EVs, an untapped source of biomarkers in liver diseases, as prognostic indicators, and combining them with existing blood biomarkers and single-nucleotide polymorphisms (NCT07185360). A clinical trial currently underway at Centre Hospitalier Universitaire de Dijon aims to differentiate normal subjects from colorectal cancer patients through the detection and characterization of circulating extracellular vesicles (EVs) in blood, including parameters such as size, concentration, and molecular composition (proteins, lipids, RNA, *etc.*) (NCT04523389).

EVs play a pivotal role in the pathogenesis and diagnosis of autoimmune diseases (Huang et al., 2025). These vesicles modulate immune responses through the transfer of bioactive molecules, such as cytokines and microRNAs, which can alter the behavior of target cells (Makhijani and McGaha, 2022). For instance, research has shown that EVs derived from immune cells carry specific miRNAs that promote inflammatory processes, thereby contributing to the development of autoimmune disorders, including systemic lupus erythematosus (SLE) and rheumatoid arthritis (Zhu et al., 2023). A study found that phosphatidylserine-negative extracellular vesicles were elevated in patients with SLE compared to healthy individuals, particularly among women and smokers (Mobarrez et al., 2016). Additionally, a prior study found that SLE patients had higher levels of CD31+/annexin V+/CD42b- EVs compared to healthy individuals, and there was a correlation between these EVs and the median overall BILAG-2004 score following treatment (Parker et al., 2014). Ding et al. found elevated levels of hcmv-miR-UL59, which is mainly contained within EVs in the plasma, in patients with oral lichen planus (OLP) (Ding et al., 2017). Another study showed increased expression of miR-4484 in salivary EVs from OLP patients and suggested this miRNA as a potential biomarker for the disease (Byun et al., 2015). Additionally, research reported varying expression levels of miR-34a-5p, miR-130b-3p, and miR-301b-3p in circulating EVs in OLP, with miR-34a-5p levels correlating with the severity of the condition (Peng et al., 2018). Moreover, the feasibility of isolating and analyzing EVs from readily accessible biofluids underscores their clinical utility, providing a promising approach for identifying novel diagnostic markers and therapeutic targets in autoimmune conditions (Zhang et al., 2020).

Extracellular vesicles have emerged as significant players in the diagnosis of infectious diseases, acting as carriers of pathogen-derived components and host immune responses (Kaparakis et al., 2010). They can encapsulate and transport microbial antigens, proteins, and nucleic acids, which can be detected in various biological fluids during infections (Zhang et al., 2020). For instance, EVs released during bacterial infections can carry virulence factors that modulate host immune responses, providing a mechanism for pathogens to evade detection (Kwaku et al., 2024). The analysis of EVs has shown promise in identifying specific biomarkers for various infectious diseases, including viral, bacterial, and parasitic infections. Recent advancements in EV isolation and characterization techniques have enhanced the sensitivity and specificity of these biomarkers, allowing for the early detection and monitoring of infectious diseases (Dominguez Rubio et al., 2022). Yoon et al. found through research that based on metagenomic analysis, five microbial patterns: *phyla Firmicutes*, *Actinobacteria, Proteobacteria*, *phyla Bacteroidetes* and *Verrucomicrobia* detected in urine EVs hold promise as potential biomarkers for the diagnosis of colorectal cancer (Yoon et al., 2023). A study involving children in Malawi found that plasma levels of EVs derived from endothelial cells were six times higher in patients with cerebral malaria (CM) compared to those with severe malaria without CM (Combes et al., 2004). This suggests a strong link between increased EV levels and the onset of CM. Additionally, EVs originating from red blood cells were shown to rise in proportion to the severity of disease in patients infected with Plasmodium falciparum and were also elevated, though to a lesser extent, in individuals infected with Plasmodium vivax and Plasmodium malariae. Importantly, antimalarial treatment led to a decrease in circulating EVs levels after 2 weeks in patients with P. vivax and P. malariae infections, but not in those with P. falciparum, indicating that sustained high EVs levels might be a marker of disease severity (Nantakomol et al., 2011). Additionally, the potential of EVs as therapeutic delivery vehicles in the context of infectious diseases is being explored, highlighting their dual role as both diagnostic and therapeutic agents (Vydrar et al., 2023). Overall, the application of EVs in infectious disease diagnostics represents a rapidly evolving field with significant implications for improving patient outcomes and disease management.

## 4 The role of extracellular vesicles in prognostic assessment

EVs have emerged as significant players in the field of prognostic assessment across various diseases, particularly in cancer, cardiovascular diseases, and neurodegenerative disorders (Hu and De, 2024; Schneider et al., 2024; AuthorAnonymous, 2024). Their ability to carry bioactive molecules allows them to reflect the physiological and pathological states of their parent cells (Salunkhe et al., 2020). This characteristic makes EVs a promising source of biomarkers for early diagnosis, monitoring disease progression, and evaluating treatment responses. The non-invasive nature of EV isolation from bodily fluids such as blood and urine enhances their utility in clinical settings, providing a window into the underlying biological processes of diseases without the need for invasive procedures (Gyorgy, 2025). The growing body of research highlights the potential of EVs to serve as reliable prognostic indicators, paving the way for personalized medicine approaches that can improve patient outcomes.

In the realm of oncology, extracellular vesicles have been identified as valuable tools for assessing cancer prognosis. For instance, studies have shown that circulating EVs can carry tumor-derived microRNAs and proteins that correlate with disease progression and patient outcomes. In melanoma, EVs derived from lymphatic drainage have been characterized to contain markers indicative of tumor progression and the presence of mutations such as BRAF ^V600E^, which are associated with a higher risk of relapse (Garcia-Silva et al., 2019). Thus, detection of the BRAF^V600E^ mutation in ES-derived EV nucleic acids could serve as a minimal residual disease/prognostic indicator, with added value over the current tissue biopsies being an almost real-time predictor of risk right after lymphadenectomy. Similarly, in cholangiocarcinoma, EVs facilitate communication between cancer cells and the tumor microenvironment, influencing disease progression and offering insights into potential therapeutic strategies (Zhang N. et al., 2023). Furthermore, in gastric cancer, the presence of specific proteins within EVs has been linked to peritoneal metastasis, demonstrating their role in predicting disease spread and patient prognosis (Li et al., 2024a). Zahra et al. demonstrated that in patients with metastatic non-small cell lung cancer, CTCs and high concentrations of PD-L1-positive small extracellular vesicles (sEVs) were significantly associated with progression-free survival (PFS) and overall survival (OS), whereas ctDNA mutations did not show a similar correlation. The integrated analysis of these biomarkers may aid in identifying patients at higher risk of poor OS outcomes. The ability to quantify these biomarkers in EVs not only enhances prognostic accuracy but also aids in the development of targeted therapies, underscoring the critical role of EVs in cancer management.

EVs have also been implicated in the risk assessment of cardiovascular diseases (CVD) (Cochain and Zernecke, 2017). They are involved in various pathophysiological processes, including inflammation, endothelial dysfunction, and thrombosis, which are central to CVD development. Research indicates that increased levels of specific EVs have been associated with hypertension and acute cardiovascular events, suggesting their potential as predictive markers (Tang et al., 2023). Moreover, EVs derived from endothelial cells have been shown to reflect the health of the vascular system, providing insights into the risk of ischemic events (Yu et al., 2025). The identification of EV-associated proteins and miRNAs linked to cardiovascular risk factors, such as obesity and metabolic syndrome, further emphasizes their role in comprehensive risk assessment strategies (Cheng et al., 2021). EVs miR-1915-3p, miR-4,507, and miR-3,656 were significantly less expressed in AMI compared to stable coronary artery disease patients, suggesting that these miRNAs might be predictive for acute myocardial infarction at an early stage (Su et al., 2020). Elevated levels of CD31^+^/Annexin V^+^ EVs are associated with an increased risk of coronary revascularization and cardiovascular mortality. These levels rise in patients who have impaired coronary artery function and cardiovascular risk factors, and they can serve as an independent predictor of cardiovascular events in individuals with stable coronary artery disease (Sinning et al., 2011). Additionally, studies have demonstrated that circulating EVs expressing CD3^+^/CD45^+^ and SMA-α^+^ are elevated in people with a high cardiovascular risk (Chiva-Blanch et al., 2016). By integrating EVs analysis into clinical practice, healthcare providers can enhance their ability to predict and manage cardiovascular risks effectively.

In neurodegenerative diseases, extracellular vesicles are gaining attention as potential biomarkers for monitoring disease progression. Conditions such as Alzheimer’s disease (AD) and Parkinson’s disease (PD) are characterized by complex pathophysiological changes that can be reflected in the cargo of EVs (Cai et al., 2024; Li et al., 2024b). For instance, studies have demonstrated that neuron-derived EVs contain proteins and RNA species that correlate with disease severity and progression in AD (Pham et al., 2024). The presence of specific microRNAs in plasma EVs has been linked to cognitive decline, highlighting their potential as early diagnostic tools (Raineri et al., 2024). Additionally, EVs can facilitate intercellular communication in the brain, contributing to the spread of pathological proteins associated with neurodegeneration (Wiersema et al., 2024). This emerging understanding positions EVs as valuable non-invasive biomarkers for tracking disease progression and therapeutic responses, offering hope for improved management strategies in neurodegenerative disorders. As research continues to elucidate the role of EVs in these conditions, their integration into clinical practice could revolutionize how we monitor and treat neurodegenerative diseases.

## 5 The potential of extracellular vesicles in treatment monitoring

**FIGURE 3:**
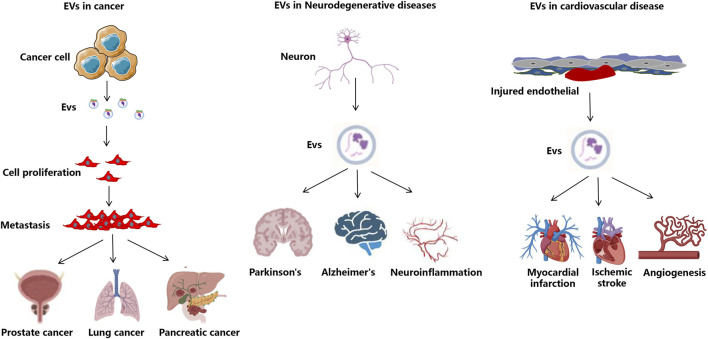
The application of EVs in disease treatment. Overview of EV roles in disease and therapeutic applications intended to attenuate cancer-related processes or to decrease tissue injury and enhance tissue repair in neurodegenerative, infectious, diabetes, cardiovascular, and kidney disease.

EVs are increasingly recognized for their potential in monitoring treatment efficacy across various medical fields, for example, tumors, neurodegenerative diseases, cardiovascular diseases, etc (Optical microscopy and transcriptomics reveal, 2024) (Figure 3). These vesicles, which are secreted by all cell types, contain a diverse array of biomolecules, making them valuable for real-time monitoring of therapeutic responses. Their presence in various bodily fluids, such as blood and urine, allows for non-invasive sampling, which is a significant advantage over traditional biopsy methods. Recent studies have demonstrated that EVs can serve as biomarkers for ongoing monitoring of treatment efficacy, particularly in cancer therapies. For instance, the dynamic changes in EV composition can reflect the biological status of tumors, providing insights into treatment responses and disease progression (Stevic et al., 2020). Moreover, the ability to analyze EVs can facilitate personalized medicine approaches, tailoring therapies based on individual patient responses.

Immunotherapy has revolutionized cancer treatment, yet its efficacy can vary significantly among patients (Ktena et al., 2024). Monitoring the effectiveness of such therapies is crucial for optimizing treatment strategies. For example, studies have shown that the presence of specific proteins and nucleic acids within EVs correlates with the response to immune checkpoint inhibitors, allowing clinicians to assess treatment effectiveness in real time (Cheng et al., 2024). The Simona study shows that circulating EVs expressing PD1 and PD-L1 predict response and mediate resistance to checkpoint inhibitors immunotherapy in metastatic melanoma (Serrati et al., 2022). Additionally, the analysis of EVs can provide insights into mechanisms of resistance, enabling timely adjustments to therapeutic regimens. The potential for EVs to serve as non-invasive indicators of immunotherapy response highlights their importance in enhancing the precision of cancer treatments (Pan et al., 2021).

The heterogeneity of cancer and individual patient responses to treatment necessitate personalized approaches to therapy (Lawrence et al., 2024). EVs can suppress tumor progression *via* modifications to the molecules expressed on their surface and inside them, independently of their origin. In line with this approach, colorectal cancer cells treated with starved tumor-cell-derived EVs loaded with miR-34a showed the inhibition of both proliferation and migration, accompanied by apoptosis, *via* the downregulation of IL-6R, STAT3, PD-L1 and VEGF-A expression *in vitro*, with prolonged survival time and impaired immune evasion in a solid tumor (Hosseini et al., 2021; Hosseini et al., 2022). Another mechanism associated with the anti-tumor activity of EVs is the sensitization to chemotherapy; for example, AT-MSC-derived EVs have been modified to express miR-122, showing that the treatment of hepatocellular carcinoma cells with these EVs increases the sensitivity of tumor cells to sorafenib (Lou et al., 2015), since miR-122 inhibits the expression of the multidrug-resistance-related genes, such as ATP-binding cassette (ABC) transporters (Xiong et al., 2021).

EVs can play a critical role in this individualized assessment by providing real-time insights into how a patient’s tumor is responding to specific drugs (Kantarci, 2023). By analyzing the molecular cargo of EVs, clinicians can gauge the effectiveness of targeted therapies and adjust treatment plans accordingly. For instance, the use of advanced technologies to profile EVs has revealed distinct signatures associated with drug resistance, enabling healthcare providers to identify which patients are likely to benefit from particular therapies. This approach not only enhances treatment efficacy but also minimizes unnecessary side effects by avoiding ineffective treatments (Lee et al., 2023). The integration of EV analysis into clinical practice represents a significant advancement in the field of personalized medicine.

Monitoring both the efficacy and potential toxicity of cancer treatments is essential for optimizing patient outcomes (Cohen et al., 2021). EVs offer a unique opportunity to achieve this dynamic monitoring through the analysis of their molecular content. Changes in the composition of EVs can indicate not only how well a treatment is working but also whether it is causing adverse effects. For example, specific biomarkers found in EVs can signal the onset of drug-related toxicities, allowing for timely interventions (Liu et al., 2023). Recent studies have demonstrated that the analysis of EVs can provide a comprehensive view of a patient’s response to therapy, capturing both therapeutic benefits and potential risks. This dual monitoring capability is particularly valuable in the context of chemotherapeutic agents, where balancing efficacy and toxicity is crucial for patient safety and treatment success (Sanchez-Manas et al., 2024). The ability to utilize EVs for real-time monitoring represents a transformative step in cancer care, enabling more informed and responsive treatment strategies.

## 6 Challenges of EVs in the field of laboratory medicine

Techniques for Isolation and Identification of Extracellular Vesicles. One of the foremost challenges in EVs research lies in the isolation and identification techniques employed. Current methods, such as ultracentrifugation and size exclusion chromatography, often yield heterogeneous populations of EVs, complicating downstream analyses and interpretations (Jia et al., 2022). The characteristics of currently widely used EVs separation methods are summarized in Table 1. The lack of standardized protocols results in variations in yield and purity, which can significantly affect the reproducibility of results across different laboratories (Royo et al., 2020). Furthermore, existing techniques may co-isolate non-EV components that can obscure the biological relevance of the findings. Emerging methods, such as microfluidic devices and immunoaffinity capture, show promise for enhancing specificity and efficiency in EV isolation (Arora et al., 2024). However, these methods are still in the developmental phase and require validation to ensure their applicability in clinical settings. The need for robust, reproducible, and standardized protocols is critical for advancing the field and facilitating the transition of EV research from bench to bedside.

**TABLE 1:** Comparison of EVs features separated by different methods.

Separation method	Principle	Yield	Purity	Repeatability	Clinical scalability
Ultracentrifuge method (UC)	Separate based on size and density	Medium	It is relatively low and easy to co-separate contaminants such as proteins	It’s okay, but it’s greatly influenced by the operator	Low, expensive equipment, and limited throughput
Density gradient centrifugation method	Separation based on EVs density	Low	Moderate, easily affected by proteins and lipids	Moderate, time-consuming	Low, not suitable for large-scale specimens
Size exclusion chromatography (SEC)	Separation is carried out based on fluid dynamics volume	Medium	Moderate, still disturbed by lipoproteins and the like	High, standardized processes	Medium, there is a risk of clogging of the chromatography column
Immunoaffinity capture method	Separation is based on surface-specific antigens	Low	High and highly specific	Moderate, affected by the batch of antibodies	Low, high cost and low throughput
Precipitation method	Polymer co-precipitates with EVs	High	It is low and easily affected by lipids and proteins	Medium, not conducive to high-precision analysis	High, low cost and high output
Target-type multi-chamber electrophoresis	Separation is carried out based on differences in size and charge ratio	High	Moderate, affected by the batch of antibodies	High potential, automated continuous separation	High, capable of high-throughput preparation
Improved chromatography	SEC binds core beads to remove soluble proteins	High	High purity, more than three times that of UC	High, standardized processes	High, suitable for large-scale clinical samples

Standardization and regulation pose significant hurdles in the clinical translation of EV-based therapies. The heterogeneity of EVs, stemming from their diverse cellular origins and biogenesis pathways, complicates the establishment of universal quality control measures (Reithmair et al., 2022). Regulatory bodies currently lack clear guidelines for the characterization and quality assessment of EVs, which can lead to inconsistencies in clinical trial outcomes and hinder the approval of EV-based therapeutics (Wang et al., 2024). Moreover, the absence of well-defined reference materials for EVs complicates the validation of methodologies used in their isolation and analysis (Wang et al., 2025). To address these challenges, it is essential to develop comprehensive guidelines that encompass the entire workflow of EV research, from isolation to characterization, to ensure that EV-based products meet the necessary safety and efficacy standards for clinical use. The quality control framework for EVs outlined in MISEV2023 can be broken down into four main steps, with a strong emphasis on creating standardized procedures and acceptance criteria for each stage: (1) Starting materials—such as cell lines and sources of biological fluids—must undergo thorough cell identity verification before EV production. Detailed records of culture conditions, including medium composition (e.g., serum presence or absence, batch numbers), cultivation environment (2D vs. 3D, use of bioreactors), and other relevant factors should be kept, as these significantly affect EV yield and molecular makeup. (2) During isolation, all key parameters related to the separation technique used must be carefully documented and reported. The entire production process should be carried out under controlled conditions that meet established quality standards. (3) Characterizing the final EV product is crucial; for clinical use, purity-measured by contaminant levels-is vital for ensuring safety and effectiveness. Adequate proof must be provided to show that contaminants have been sufficiently removed or minimized to acceptable levels. (4) Functional testing and stability monitoring: Functional assays, either *in vitro* or *in vivo*, should be designed based on the intended therapeutic use of the EVs (such as immune modulation, tissue repair, or drug delivery). Moreover, the physical integrity, surface marker profiles, and biological activity of EVs should be regularly checked during storage to guarantee consistent performance.

The clinical translation of EVs as therapeutic agents faces several barriers, including technical, biological, and regulatory challenges. Despite the promising therapeutic potential of EVs, particularly in areas such as cancer and neurodegenerative diseases, their clinical application is often impeded by issues related to scalability, reproducibility, and the complexity of their biological effects (Li et al., 2025). Furthermore, the intricate mechanisms by which EVs exert their effects are not yet fully understood, which complicates the development of targeted therapies (Song et al., 2024). However, ongoing research is exploring innovative strategies to enhance the therapeutic efficacy of EVs, such as engineering modifications to improve targeting and cargo delivery (Wang et al., 2024). The integration of EVs into existing treatment paradigms, combined with advancements in nanotechnology and drug delivery systems, holds promise for overcoming these barriers. As the field matures, collaborative efforts among researchers, clinicians, and regulatory agencies will be pivotal in realizing the full potential of EVs in clinical practice.

## 7 The development trend of EVs in the field of laboratory medicine

Multi-omics integration and big data analysis. As a marker carrier at the subcellular scale, EVs carry a variety of biomolecules such as proteins, RNA, DNA, and metabolites, etc (Yokoi et al., 2025). Bollard and colleagues discovered that analyzing plasma-derived extracellular vesicles using both proteomics and metabolomics can be an effective diagnostic tool for melanoma, achieving a classification accuracy of 85.11% when distinguishing melanoma patients from healthy individuals (Bollard et al., 2024). For instance, researchers recently showed that measuring both α-synuclein and clusterin together in serum L1CAM-positive EVs was very effective (AUC = 0.98) at distinguishing Parkinson’s disease from atypical parkinsonism. This finding was based on 735 samples from four separate groups and outperformed the accuracy of each individual marker alone, which had AUC values around 0.82 to 0.86 (Jiang et al., 2020). Using multi-omics integration for the joint analysis of different types of markers, and solving the technical problems of complex data analysis with the help of the big data accumulated from basic research and clinical practice, and artificial intelligence to construct a multi-dimensional disease prediction and diagnostic model, we can not only mine potential markers of EVs in the high-throughput multi-dimensional genomic data to predict their diagnostic value in different disease groups, but also reveal biological features that are difficult to be found by traditional methods, thus significantly improving the diagnostic specificity and sensitivity of EVs markers (Miceli et al., 2024). It can not only mine potential markers in EVs from high-throughput multidimensional histological data and predict their diagnostic value in different disease groups, but also reveal biological features that are difficult to be found by traditional methods, thus significantly improving the diagnostic specificity and sensitivity of EVs markers, which is the future direction of the development of EVs and its application prospects (Yin et al., 2024).

Development of clinically appropriate technology for the isolation and detection of EVs. With the rapid advancement of cross-disciplinary medicine, emerging technologies such as nanotechnology, microfluidics, super-resolution microscopy, functional materials, and artificial intelligence have been progressively integrated into the experimental platforms for EVs. These innovations have substantially enhanced the separation efficiency, detection sensitivity, and specificity of EVs (Chen et al., 2021). For instance, ultrasonic nanofiltration technology leverages the synergistic advantages of ultrasound and nanofiltration membranes to efficiently purify EVs within a short timeframe, making it suitable for large-scale clinical samples (Chen et al., 2021). Ultrasensitive flow cytometry and droplet microfluidics enable highly sensitive detection of EVs (Khanna et al., 2023; Meng et al., 2023), while super-resolution microscopy is utilized for characterizing EV subpopulations and analyzing their interactions with cells (Zhang Z. et al., 2023). Given the complexity of body fluid samples and the diversity and high heterogeneity of EV markers, further efforts are required in three key areas: automated analysis of EV isolation and detection platforms, high-precision detection platforms for individual EVs, and high-throughput multi-marker detection platforms (Feng et al., 2025). Such advancements are essential for translating EV research into clinical applications. Continuous innovation and optimization of EV isolation and detection technologies are necessary to improve detection sensitivity and specificity, reduce detection time, lower costs, and facilitate large-scale clinical screening and research on EVs (Ma et al., 2019). Ultimately, these improvements aim to establish EV isolation and detection as a practical and reliable technology, providing advanced tools for health management, disease prediction, early diagnosis, condition assessment, treatment guidance, and therapeutic efficacy monitoring.

Standardized and normalized quality system construction. A robust quality control system is critical for ensuring the reliability and consistency of test results. Establishing a reference material production, research, and quality assurance system constitutes the core of this endeavor. First, EV reference materials must achieve standardization and reproducibility, stable physicochemical properties, well-defined biological characteristics, quantifiability, and ease of acquisition-these are also the primary objectives of EV research (Vogel et al., 2021). Second, the EV testing process encompasses multiple steps, from sample collection and processing to EV isolation, testing, and data analysis. Each step may introduce errors and variations that could compromise result accuracy. To address these challenges, ISEV and the Committee on Extracellular Vesicle Research and Application (CSEV) of the Chinese Society of Research Hospitals have developed a series of position papers and quality control procedures aimed at monitoring and mitigating errors and variability in EV testing. For instance, MISEV2023, the latest guideline for EV research issued by ISEV, provides detailed specifications for experimental practices and data reporting based on cutting-edge scientific advancements and expert consensus (Author Anonymous, 2024). Additionally, the MIBlood-EV Quality Control Reporting Framework was established to encompass pre-analytical variables and quality control methods for blood samples, thereby promoting standardization and enabling cross-laboratory comparisons (Lucien et al., 2023). For instance, the U.S. Food and Drug Administration (FDA) has issued a series of guidance documents on liquid biopsy and extracellular vesicle (EV) markers, outlining specific requirements for clinical trial design, data submission, and approval processes. In 2016, ExoDx Prostate received regulatory clearance from the U.S. FDA, becoming the first prostate cancer risk assessment tool based on EVs’s RNA (McKiernan et al., 2016).

## 8 Future perspectives

The 2013 Nobel Prize in Physiology or Medicine was awarded to American scientists James E. Rothman and Randy W. Schekman, along with German scientist Thomas C. Südhof, in recognition of their discovery of the regulatory mechanisms governing intracellular vesicle transport. This breakthrough also ignited a global surge of research into EVs. As a promising biomarker, EVs show vast potential for development within laboratory medicine. However, for EVs to be fully integrated into clinical practice as diagnostic and therapeutic tools, ongoing efforts are required in areas such as multi-omics integration and big data analysis, the creation of clinically suitable separation and detection technologies, the establishment of standardized quality control systems, as well as clinical trials and regulatory approvals. Given the unique advantages of EVs and the rapid advancement of related technologies, through the dedicated work of scientists, clinicians, and laboratory professionals, EVs are expected to play a significant role in clinical diagnosis and treatment in the future, ultimately enhancing human health.
